# Schwannoma of the brachial plexus; report of two cases involving the C7 root

**DOI:** 10.1186/1749-7221-8-12

**Published:** 2013-11-04

**Authors:** Mamoon Rashid, Omer Salahuddin, Shumaila Yousaf, Uzair A Qazi, Kanwal Yousaf

**Affiliations:** 1 Shifa International Hospital, Islamabad, Pakistan

**Keywords:** Brachial plexus tumors, Schwannoma, Supraclavicular swelling

## Abstract

Brachial plexus schwannomas are rare tumors. They are benign nerve sheath tumors and only about 5% of Schwannoma arise from the brachial plexus. They pose a great challenge to surgeons due to their rare occurrence and complex anatomical location. We present two cases who presented with a supraclavicular swelling, that were proven to be schwannoma on histopathology.

## Introduction

Schwannomas are relatively rare tumors and a high proportion of these develop in the head and neck [1]. They usually involve cranial nerves and the sympathetic chain with relative sparing of the brachial plexus [2]. Benign schwannomas are the commonest peripheral nerve tumors and malignant transformation is extremely rare. These tumors are composed of Schwann cells and are neuroectodermal in origin [3]. The tumor is eccentric and the major nerve trunk is placed peripherally which is one of the diagnostic criteria. Patients usually present to seek treatment because of pain, loss of function, numbness or a progressively growing mass in the supraclavicular region [4]. The diagnosis is usually clinical but can be easily misdiagnosed as enlarged supraclavicular lymph node. We present two cases of Schwannoma of the brachial plexus involving C7 nerve root one of which was initially misdiagnosed.

### Case Report

#### Case 1

**Figure 1:**
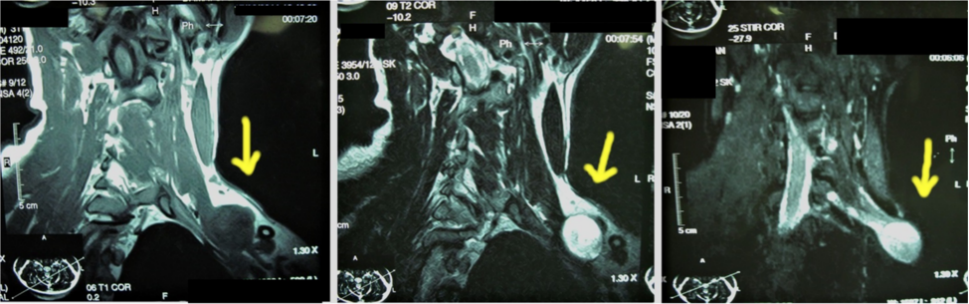
MRI neck showing well circumscribed lesion involving C7 root with enhancement on T2 weighted images.

A 32 year old male presented with a painless swelling in his left supraclavicular region for the last 2 years. It had progressively enlarged from the size of an almond to that of a lime. There was no history of trauma, fever or systemic illness. He only complained of a visible swelling but had no weakness, numbness or loss of function of the upper limb. On examination, he had a 3.5 x 3.0 cm firm, mobile, tender swelling in his left supraclavicular region. Muscle power in all muscles was 5/5, sensations were intact and there were no signs of wasting. Tinel sign was positive with tingling sensation along the shoulder tip. FNAC showed spindle shaped cells and Schwan cells and a diagnosis of schwannoma was made. MRI neck showed a diffuse swelling involving the lower trunk of Brachial plexus. The tumor showed same intensity as skeletal muscle on T1 weighted images but increased signals on T2 weighted images (Figure 1).

**Figure 2:**
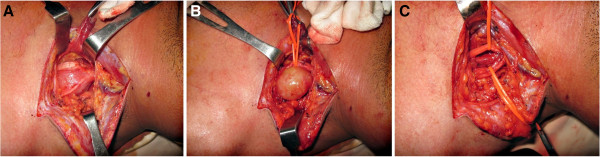
**Intraoperative details of schwannoma brachial plexus C7 root in a 32 year male. (A)** Well encapsulated mass splitting the fascicles of C7 root, **(B)** schwannoma after careful separation from fasciles. **(C)** Nerve fascicles after enucleation.

The surgery was performed under general anesthesia and lazy S supraclavicular incision was made to expose the tumor. Anterior scalenotomy was performed to improve the exposure of the roots and trunks. Per-operatively, a well encapsulated mass was found lying centrally within the C7 root and was pushing the nerve fascicles towards the periphery (Figure 2). The tumor was carefully enucleated avoiding any damage to the trunk. The histopathology of the tumor was consistent with Schwannoma. In the post-operative period, the patient had an uneventful recovery with complete range of active motion and intact sensations. He was closely followed up with monthly visits for 6 months and remained symptom free during this period. In a subsequent visit 18 months after surgery, he was back to his normal routine and there was no evidence of recurrence.

#### Case 2

**Figure 3:**
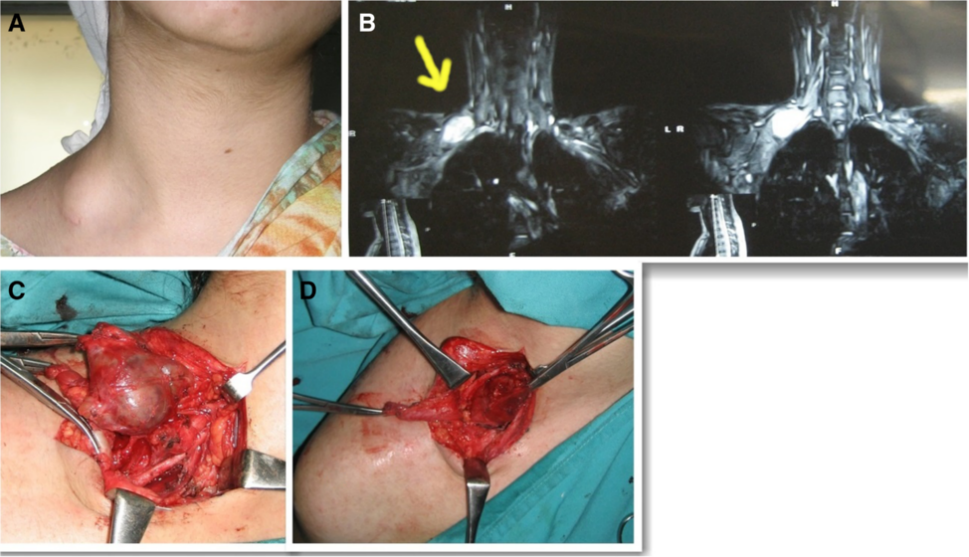
(A) Shwannoma right Supraclavicular region, (B) MRI showing T2 weighted image enhancement, (C) peroperative enucleation, (D) After enucleation.

A 23 year old lady was referred to the plastic surgery department with complaints of a painful swelling in the right supraclavicular region of 2 months duration (Figure 3) and loss of shoulder abduction and weak elbow flexion of 4 weeks duration. She had previously undergone an attempt of an excisional biopsy of the same swelling 4 weeks before which had possibly resulted in damage to C5 and C6 nerve roots. There was no history of any functional or sensory deficit of right upper limb before surgery. The histopathology report of the previous excision biopsy showed unremarkable adipose tissue only. MRI with contrast showed a large lesion encasing the C7 root of the right brachial plexus (Figure 3).

Anterior cervical approach incorporating the previous incision was used for exploration and excision of the lesion. Exploration revealed a lesion involving the C7 root and severed C5 and C6 nerve roots. Encapsulated tumor was enucleated with preservation of C7 root. (Figure 3) C5 and C6 nerve roots were repaired by sural nerve cable grafts. Histopathology was consistent with Schwannoma. Post operative recovery was uneventful with good recovery of shoulder abduction and elbow flexion within one year.

## Discussion

Brachial plexus tumors are uncommon and comprise of only 5% tumors of the upper limb [5]. Schwannomas are benign well encapsulated tumors arising from the nerve sheath [6]. Schwannomas usually present with local slow growing mass but may present with symptoms of nerve compression. Grossly these tumors are round, oval or plexiform and may appear yellow or gray [7]. They may present at all ages but most commonly occur at second to fourth decade of life [8]. Our cases also presented in second and third decades of life.

The diagnosis of Schwannoma arising in the brachial plexus or the upper limb can be diagnosed clinically on following points; A firm non-tender mobile swelling with symptoms of pain and percussion producing paraesthesias along the nerve involved. MRI can sometimes be very helpful in confirmation of diagnosis. Mostly it presents as a well encapsulated solitary lesion causing displacement of the fascicles [9,10].

Surgery is indicated for tumors causing neurological deficit, discomfort, progressively growing lesions with a suspicion of malignancy and to prevent or minimize neural damage [7]. Complete resection of these tumors with preservation of surrounding nerves should be the goal. Neural fascicles surrounding the Schwannoma are usually separable and enucleation of the tumor is almost always possible [3,10]. In our case we were also able to dissect the nerve fascicles and successfully enucleate the tumor with preservation of all the nerve fascicles.

## Conclusion

Schwannoma of the brachial plexus region is a rare entity. Proper diagnosis of the lesion must be established before surgery as it can be easily mistaken as enlarged supraclavicular lymph node as happened in one of our cases and can result in an iatrogenic injury. Schwannoma should be included in the differential diagnosis of supraclavicular swellings and MRI should be performed if there is any suspicion.

### Consent

Written informed consent was obtained from the patients for publication of this Case report and any accompanying images. A copy of the written consent is available for review by the Editor-in-Chief of this journal. Furthermore an approval from institutional review board was also taken from “Institutional Review Board and Ethics Committee, Shifa International Hospital, Shifa College of Medicine and Shifa College of Nursing” and can be sent on demand.

## Competing interests

The authors declare that they have no competing interests. 

## Authors’ contributions

MR final proof reading and final approval for publication. OS conception, design and final drafting. SY initial drafting and acquisition of data. UAQ Acquisition of images, literature search, proof reading. KY drafting and critical revision. All authors read and approved the final manuscript.
